# Risk Factors, Symptomatology, and Predictors of Mortality Among COVID-19 Inpatients Presenting With Delirium Symptoms in a Tertiary Hospital in the Philippines

**DOI:** 10.1192/bjo.2024.700

**Published:** 2024-08-01

**Authors:** Sedric John Factor, Josefina Ly-Uson, Katrina Joy Aligam, Marie Angelique Gelvezon

**Affiliations:** University of the Philippines - Philippine General Hospital, Manila, Philippines

## Abstract

**Aims:**

The prevalence of delirium among confirmed COVID-19 patients is around 12–33%. Delirium in COVID-19 patients is associated with worse functional outcomes; and associated with length of hospital stay, admission to ICU, and ventilator utilization. COVID-19 patients with delirium have a significantly higher risk for mortality than those who did not develop delirium. This study aimed to describe the risk factors, symptomatology, and predictors of mortality of COVID-19 patients presenting with delirium symptoms admitted between January and October 2021 to the Philippine General Hospital, a public tertiary hospital in the Philippines.

**Methods:**

Medical records of adult COVID-19 patients admitted to the Philippine General Hospital were analyzed. Descriptive statistics were used to summarize the demographic and clinical history. Univariate and multivariate logistic regression were done to determine the variables that predict mortality.

**Results:**

One in five (20.01%) COVID-19 patients presented with delirium; of the 1,992 medical records reviewed, 400 patients had either presented with symptoms of delirium or were diagnosed with delirium.

Of the 400 patients, 36.5% were not diagnosed with delirium, only 7% were referred to Psychiatry, and 74% expired during admission. Patients referred to Psychiatry had lower mortality odds than those not referred (aOR = 0.069, p = 0.014). Before the COVID-19 pandemic, patients with psychiatric symptoms from organic causes are already less likely to be referred to psychiatrists. Furthermore, studies have shown that delirium is under-recognized among patients with COVID-19. Early referral to a psychiatrist for assessment and management may possibly be protective against mortality.

Those who received midazolam had higher odds of mortality (aOR = 3.112, p = 0.001). Currently, no literature supports the association between midazolam use and mortality among COVID-19 patients with delirium; however, it is known that midazolam use puts patients at increased risk for delirium and mortality.

Patients with decreased sensorium (aOR = 7.438) and decreased psychomotor activity (aOR = 3.857) had higher odds of mortality (p < 0.001). Decreased sensorium and decreased psychomotor activity are typical in patients with hypoactive delirium; hypoactive delirium is a known prognosticator for patient mortality. The only available studies on specific delirium symptomatology show that decreased sensorium and decreased psychomotor activity are common among COVID-19 patients with delirium.

**Conclusion:**

Timely assessment and appropriate management are critical for COVID-19 patients with delirium symptoms, especially those at an increased risk for mortality. Clinicians dealing with COVID-19 patients presenting with delirium must be reoriented to delirium symptomatology, initial interventions, and indications for referral to psychiatrists.

